# Supplementary material for: Bias-corrected climate projections for South Asia from Coupled Model Intercomparison Project-6
Source: Sci Data. 2020 Oct 12;7:338. doi: 10.1038/s41597-020-00681-1 (PMC7550601; doi:10.1038/s41597-020-00681-1)
Supplement: Supplementary file 1 — Supplementary Information [file 41597_2020_681_MOESM1_ESM.docx]

**Supplemental Information**

**Bias-corrected climate projections for South Asia from Coupled Model Intercomparison Project-6**

Vimal Mishra^1,2^, Udit Bhatia^1^, Amar Deep Tiwari^1^

1--Civil Engineering, Indian Institute of Technology (IIT) Gandhinagar

2—Earth Sciences, Indian Institute of Technology Gandhinagar

^*^Corresponding Author: [vmishra@iitgn.ac.in](mailto:vmishra@iitgn.ac.in)

**Table of contents**

| **Figure/Table** | **Page No.** |
| --- | --- |
| Fig. S1 | 2 |
| Fig. S2 | 3 |
| Fig. S3 | 3 |
| Fig S4 | 4 |
| Table S1 | 5 |
| Table S2 | 6 |
| Table S3 | 7 |
| Table S4 | 8 |
| Table S5 | 9-10 |
| Table S6 | 11 |
| Table S7 | 12 |

**
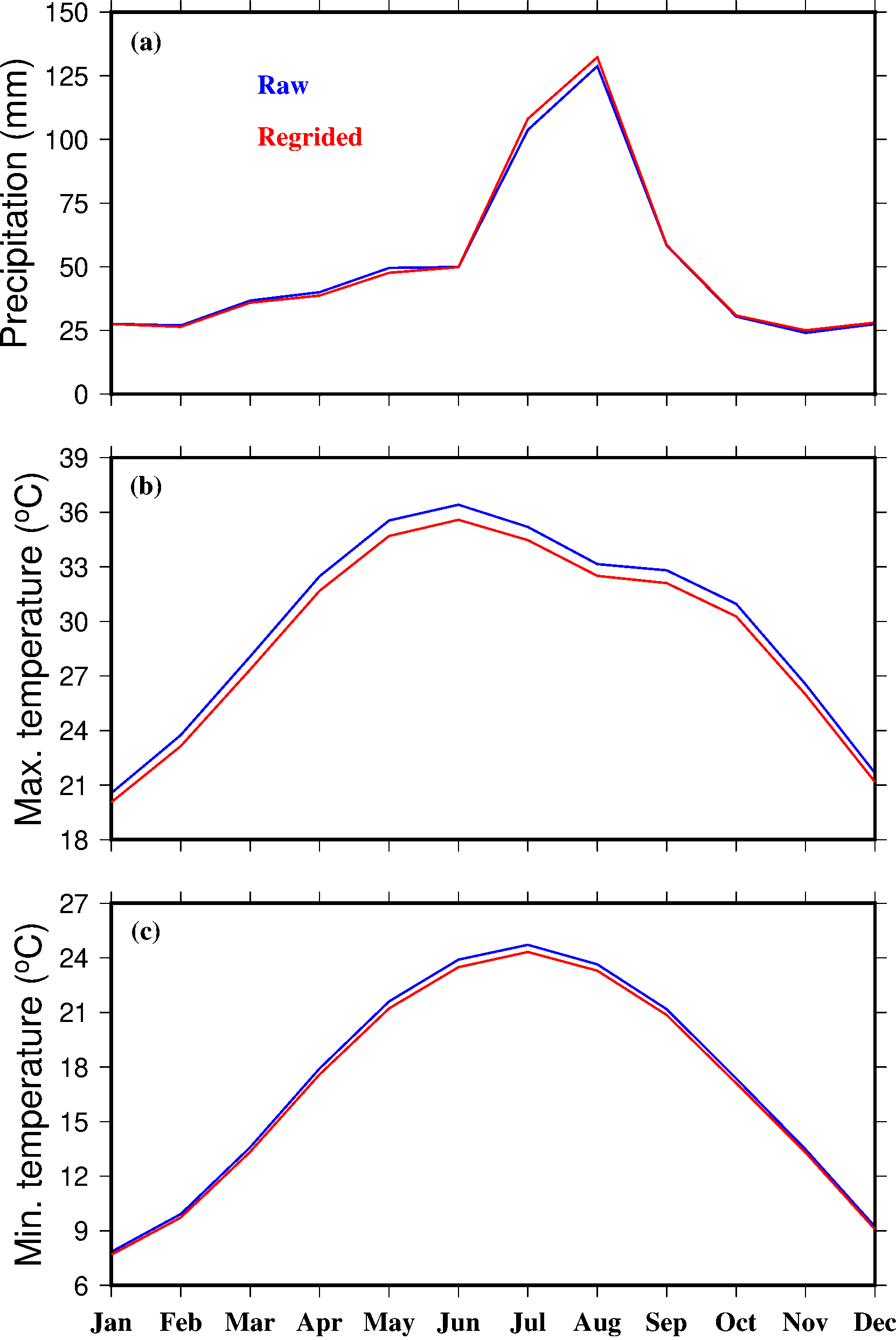
**

Figure S1. Seasonal cycle of raw (ACCESS-CM2 at 1.25 lat x 1.875 degree long) and regrided at (0.25 x 0.25 degree) (a) precipitation, (b) max. Temperature, and (c) min. Temperature for the 1951-2014 period.

**Figure S2:** Illustration of Quantile-Quantile mapping of model output ($x_{m}$) (obtained from CMIP6 archive) and observed daily maximum temperature$\left( x_{o} \right)$ over a representative grid-point chosen randomly from the Indian Subcontinent. While at 20^th^ percentile ($\phi_{0.2}$), $x_{m}$ is lower than $x_{o}$ for the same quantile, $x_{m}$ exhibits higher bias than $x_{o}$ at 60^th^ percentile ($\phi_{0.6})$. The empirical adjustment of modeled outputs using non-parametric quantile mapping results in the elimination of systematic bias between $x_{m}$ and $x_{o}$.


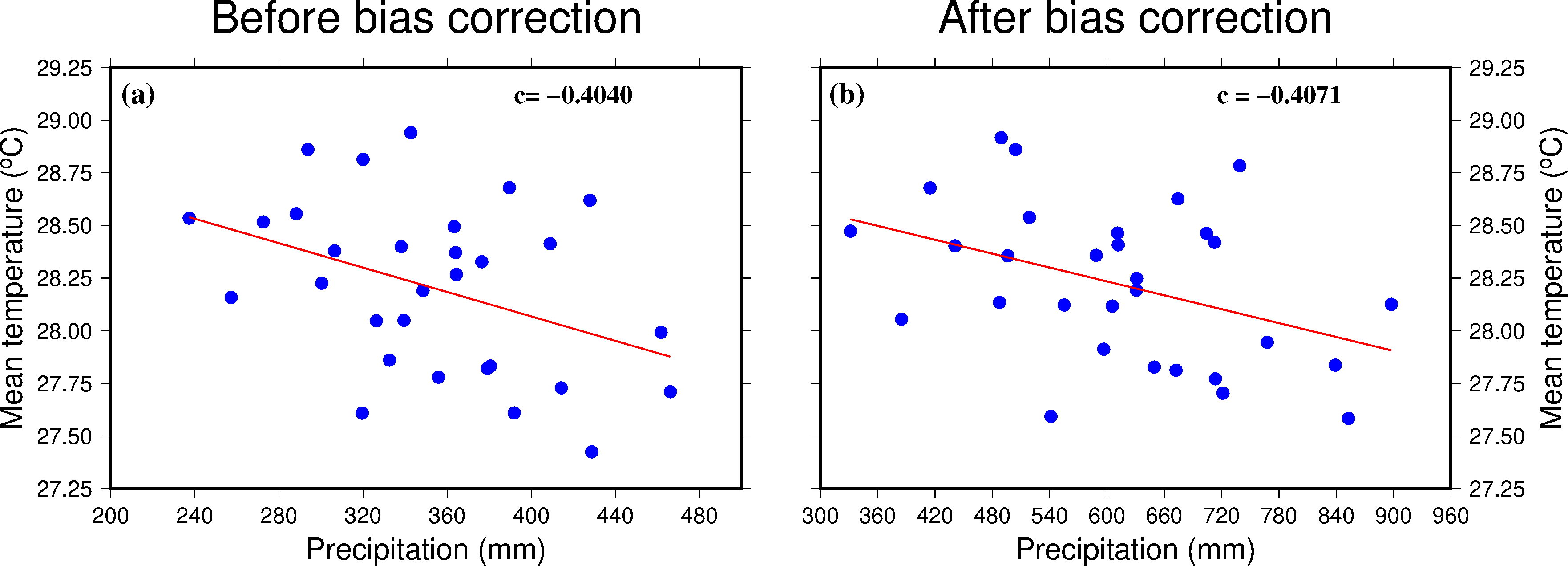


Figure S3: (a) Correlation between the monsoon season precipitation and mean temperature of the ACCESS-CM2 model at 0.25 degrees (a) before bias correction and (b) after bias correction for 1971-2000 period. The red line represents the regression line.


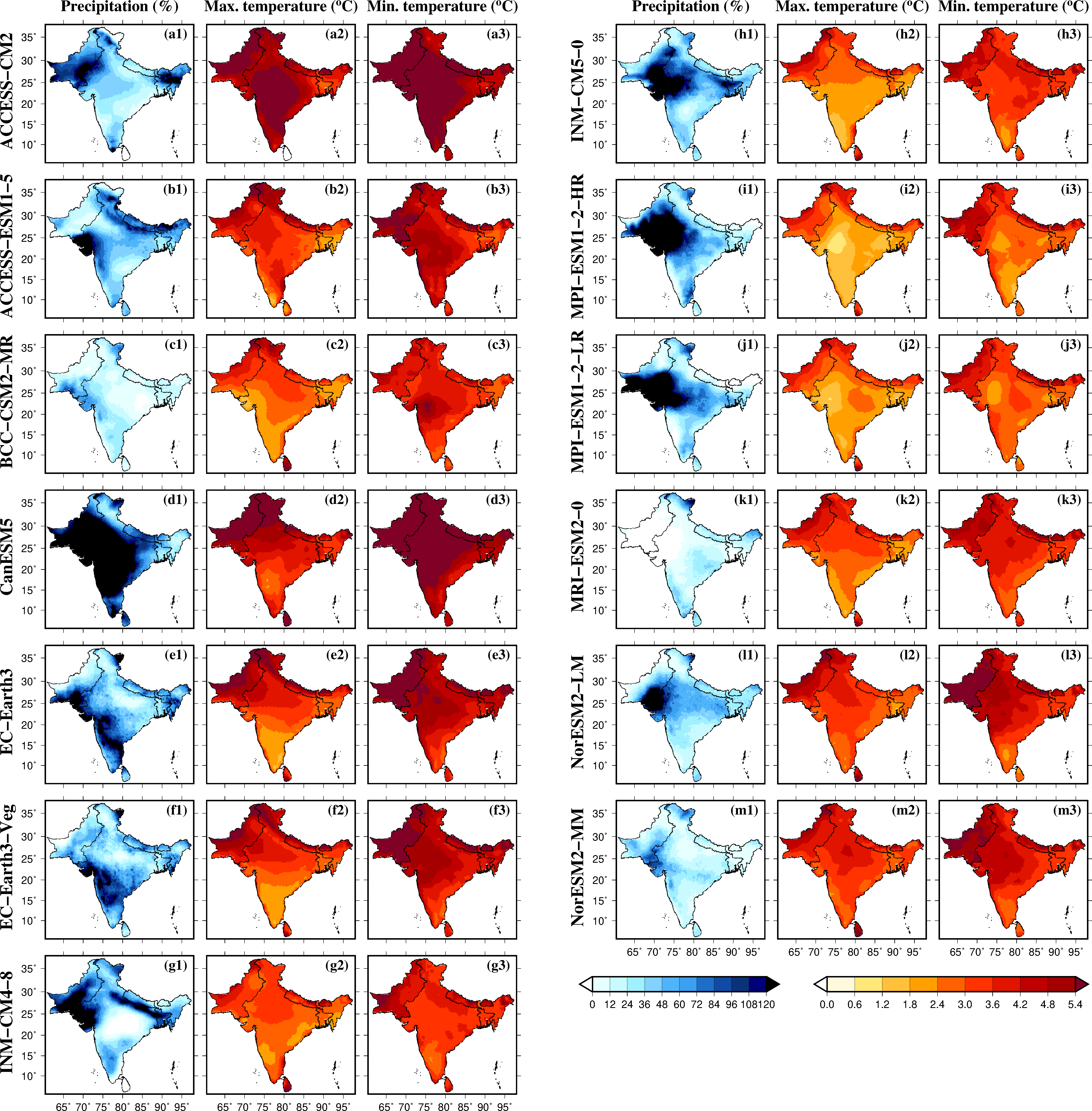


Figure S4: (a1-a3) The projected change in in mean annual precipitation (%), mean annual maximum temperature (°C), and mean annual minimum temperature (°C) for the Far (2074-2100) period with respect to the historical period (1988-2014) from the individual GCMs.

Table S1. CMIP6 GCMs that are used for bias corrected projections.

| **S. No.** | **Model name** | **Latitude resolution (degree)** | **Longitude resolution (degree)** |
| --- | --- | --- | --- |
| 1 | ACCESS-CM2 | 1.25 | 1.875 |
| 2 | ACCESS-ESM1-5 | 1.25 | 1.875 |
| 3 | BCC-CSM2-MR | 1.1215 | 1.125 |
| 4 | CanESM5 | 2.7906 | 2.8125 |
| 5 | EC-Earth3 | 0.7018 | 0.703125 |
| 6 | EC-Earth3-Veg | 0.7018 | 0.703125 |
| 7 | INM-CM4-8 | 1.5 | 2 |
| 8 | INM-CM5-0 | 1.5 | 2 |
| 9 | MPI-ESM1-2-HR | 0.9351 | 0.9375 |
| 10 | MPI-ESM1-2-LR | 1.8653 | 1.875 |
| 11 | MRI-ESM2-0 | 1.1215 | 1.125 |
| 12 | NorESM2-LM | 1.8947 | 2.5 |
| 13 | NorESM2-MM | 0.9424 | 1.25 |

Table S2: Country wise multimodel ensemble mean projected change in mean annual precipitation (%). Historical mean is provided in mm. Uncertainty (one standard deviation) was estimated using bias corrected data from 13 CMIP6-GCMs.

| **Country** | | Bangladesh | Bhutan | India | Nepal | Pakistan | Sri Lanka |
| --- | --- | --- | --- | --- | --- | --- | --- |
| **Historic mean (mm)** | | 2424.62 | 1921.94 | 1134.79 | 1394.00 | 290.84 | 1741.22 |
| **ssp126** | **Near** | 6.20 ± 9.22 | 3.14 ± 6.65 | 11.40 ± 8.26 | 7.46 ± 7.31 | 19.94 ± 10.28 | 5.32 ± 12.09 |
|  | **Mid** | 10.92 ± 10.52 | 6.65 ± 10.20 | 16.09 ± 8.82 | 11.27 ± 7.40 | 18.55 ± 12.56 | 11.51 ± 12.70 |
|  | **Far** | 9.98 ± 7.19 | 11.43 ± 9.81 | 16.13 ± 9.12 | 13.25 ± 6.48 | 17.16 ± 14.01 | 11.13 ± 12.63 |
| **ssp245** | **Near** | 3.71 ± 9.68 | 0.67 ± 8.79 | 7.39 ± 6.40 | 4.60 ± 6.83 | 13.00 ± 13.32 | 6.48 ± 9.08 |
|  | **Mid** | 10.92 ± 13.69 | 6.88 ± 12.42 | 16.24 ± 10.23 | 12.02 ± 10.46 | 17.53 ± 15.01 | 14.19 ± 14.12 |
|  | **Far** | 15.05 ± 15.38 | 11.53 ± 13.93 | 22.80 ± 12.23 | 15.16 ± 11.98 | 27.74 ± 17.65 | 18.84 ± 16.23 |
| **ssp370** | **Near** | 3.66 ± 7.28 | 2.19 ± 9.56 | 9.76 ± 7.63 | 6.63 ± 8.19 | 17.68 ± 15.66 | 7.82 ± 11.20 |
|  | **Mid** | 11.68 ± 13.48 | 7.76 ± 15.19 | 17.94 ± 15.69 | 13.59 ± 15.55 | 24.43 ± 18.01 | 12.73 ± 18.77 |
|  | **Far** | 27.02 ± 20.00 | 20.85 ± 22.63 | 34.53 ± 20.35 | 26.42 ± 18.28 | 45.86 ± 28.64 | 23.83 ± 20.89 |
| **ssp585** | **Near** | 6.19 ± 10.54 | 0.91 ± 9.71 | 9.78 ± 10.21 | 5.48 ± 7.41 | 20.44 ± 11.87 | 6.50 ± 14.01 |
|  | **Mid** | 18.11 ± 16.05 | 12.08 ± 13.79 | 24.30 ± 17.81 | 18.38 ± 12.78 | 31.98 ± 27.75 | 20.02 ± 19.87 |
|  | **Far** | 39.46 ± 25.37 | 35.29 ± 33.75 | 48.67 ± 28.66 | 39.96 ± 26.13 | 53.58 ± 42.50 | 31.79 ± 23.12 |

Table S3: Country wise multimodel projected change in mean annual maximum temperature (°C). Uncertainty (one standard deviation) was estimated using bias corrected data from 13 CMIP6-GCMs.

| **Country** | | Bangladesh | Bhutan | India | Nepal | Pakistan | Sri Lanka |
| --- | --- | --- | --- | --- | --- | --- | --- |
| **Historic mean** | | 30.35 | 17.01 | 30.67 | 19.93 | 29.44 | 30.61 |
| **ssp126** | **Near** | 0.48 ± 0.18 | 0.84 ± 0.17 | 0.58 ± 0.24 | 0.82 ± 0.22 | 0.90 ± 0.26 | 0.97 ± 0.37 |
|  | **Mid** | 0.90 ± 0.17 | 1.30 ± 0.31 | 1.02 ± 0.38 | 1.28 ± 0.35 | 1.35 ± 0.42 | 1.46 ± 0.60 |
|  | **Far** | 0.95 ± 0.24 | 1.31 ± 0.40 | 1.05 ± 0.40 | 1.32 ± 0.45 | 1.39 ± 0.46 | 1.49 ± 0.64 |
| **ssp245** | **Near** | 0.33 ± 0.27 | 0.82 ± 0.16 | 0.56 ± 0.27 | 0.81 ± 0.21 | 0.94 ± 0.25 | 0.97 ± 0.44 |
|  | **Mid** | 1.00 ± 0.26 | 1.66 ± 0.31 | 1.28 ± 0.44 | 1.64 ± 0.34 | 1.80 ± 0.45 | 1.97 ± 0.74 |
|  | **Far** | 1.42 ± 0.32 | 2.20 ± 0.45 | 1.72 ± 0.54 | 2.18 ± 0.45 | 2.36 ± 0.58 | 2.59 ± 0.96 |
| **ssp370** | **Near** | 0.29 ± 0.31 | 0.81 ± 0.23 | 0.48 ± 0.36 | 0.77 ± 0.28 | 0.91 ± 0.34 | 1.03 ± 0.45 |
|  | **Mid** | 0.95 ± 0.40 | 1.86 ± 0.30 | 1.37 ± 0.49 | 1.81 ± 0.36 | 2.05 ± 0.54 | 2.45 ± 0.93 |
|  | **Far** | 1.85 ± 0.53 | 3.18 ± 0.58 | 2.40 ± 0.70 | 3.02 ± 0.57 | 3.31 ± 0.74 | 4.20 ± 1.59 |
| **ssp585** | **Near** | 0.36 ± 0.39 | 0.97 ± 0.20 | 0.61 ± 0.35 | 0.91 ± 0.29 | 1.07 ± 0.33 | 1.13 ± 0.46 |
|  | **Mid** | 1.45 ± 0.41 | 2.42 ± 0.39 | 1.81 ± 0.54 | 2.31 ± 0.45 | 2.55 ± 0.55 | 2.95 ± 1.16 |
|  | **Far** | 2.64 ± 0.55 | 4.22 ± 0.75 | 3.22 ± 0.88 | 3.97 ± 0.77 | 4.33 ± 0.98 | 5.34 ± 2.05 |

Table S4: Same as Table S3 but for mean annual minimum temperature. Uncertainty (one standard deviation) was estimated using bias corrected data from 13 CMIP6-GCMs.

| **Country** | | Bangladesh | Bhutan | India | Nepal | Pakistan | Sri Lanka |
| --- | --- | --- | --- | --- | --- | --- | --- |
| **Historic mean** | | 21.00 | 5.66 | 18.68 | 7.57 | 15.52 | 23.31 |
| **ssp126** | **Near** | 0.69 ± 0.20 | 1.15 ± 0.28 | 0.84 ± 0.29 | 1.12 ± 0.29 | 1.27 ± 0.39 | 0.82 ± 0.23 |
|  | **Mid** | 1.11 ± 0.27 | 1.61 ± 0.48 | 1.25 ± 0.45 | 1.55 ± 0.45 | 1.67 ± 0.53 | 1.15 ± 0.35 |
|  | **Far** | 1.14 ± 0.29 | 1.58 ± 0.55 | 1.25 ± 0.48 | 1.54 ± 0.51 | 1.65 ± 0.59 | 1.13 ± 0.37 |
| **ssp245** | **Near** | 0.65 ± 0.33 | 1.20 ± 0.27 | 0.84 ± 0.33 | 1.14 ± 0.30 | 1.28 ± 0.38 | 0.85 ± 0.24 |
|  | **Mid** | 1.41 ± 0.37 | 2.24 ± 0.49 | 1.68 ± 0.50 | 2.10 ± 0.51 | 2.32 ± 0.60 | 1.54 ± 0.37 |
|  | **Far** | 1.88 ± 0.42 | 2.93 ± 0.52 | 2.19 ± 0.63 | 2.73 ± 0.57 | 2.97 ± 0.80 | 1.91 ± 0.45 |
| **ssp370** | **Near** | 0.68 ± 0.36 | 1.28 ± 0.36 | 0.82 ± 0.36 | 1.17 ± 0.39 | 1.30 ± 0.44 | 0.89 ± 0.25 |
|  | **Mid** | 1.64 ± 0.51 | 2.75 ± 0.57 | 1.96 ± 0.62 | 2.53 ± 0.66 | 2.72 ± 0.77 | 1.81 ± 0.43 |
|  | **Far** | 2.78 ± 0.64 | 4.41 ± 0.73 | 3.25 ± 0.87 | 4.06 ± 0.90 | 4.38 ± 1.14 | 2.82 ± 0.61 |
| **ssp585** | **Near** | 0.71 ± 0.46 | 1.34 ± 0.35 | 0.96 ± 0.39 | 1.29 ± 0.38 | 1.50 ± 0.47 | 0.99 ± 0.26 |
|  | **Mid** | 2.06 ± 0.43 | 3.15 ± 0.57 | 2.43 ± 0.65 | 3.03 ± 0.64 | 3.35 ± 0.86 | 2.16 ± 0.46 |
|  | **Far** | 3.56 ± 0.68 | 5.30 ± 0.90 | 4.14 ± 1.03 | 5.06 ± 1.08 | 5.52 ± 1.44 | 3.47 ± 0.70 |

**Table S5:** Basin wise multimodel ensemble mean projected change in mean annual precipitation (%)

Uncertainty (one standard deviation) was estimated using bias corrected data from 13 CMIP6-GCMs.

| **Basin** | **Historic mean (mm)** | **ssp126** | | | **ssp245** | | | **ssp370** | | | **ssp585** | | |
| --- | --- | --- | --- | --- | --- | --- | --- | --- | --- | --- | --- | --- | --- |
|  |  | **Near** | **Mid** | **Far** | **Near** | **Mid** | **Far** | **Near** | **Mid** | **Far** | **Near** | **Mid** | **Far** |
| Bhahmani | 1452.06 | 8.11 ± 9.77 | 14.44 ± 9.79 | 16.95 ± 9.78 | 2.24 ± 5.95 | 9.84 ± 5.73 | 16.60 ± 11.26 | 3.16 ± 8.16 | 8.10 ± 13.52 | 20.57 ± 14.45 | 3.95 ± 9.02 | 16.95 ± 12.99 | 33.13 ± 16.99 |
| Brahmaputra | 1692.12 | 4.18 ± 5.06 | 8.47 ± 7.65 | 11.15 ± 7.50 | 2.15 ± 7.23 | 9.44 ± 8.74 | 13.47 ± 9.33 | 3.56 ± 6.60 | 10.90 ± 9.88 | 25.91 ± 15.16 | 3.22 ± 6.15 | 15.89 ± 9.56 | 39.04 ± 22.11 |
| Cauvery | 942.30 | 8.33 ± 11.95 | 14.53 ± 10.93 | 12.95 ± 10.89 | 7.42 ± 8.38 | 15.71 ± 10.76 | 19.08 ± 12.20 | 8.12 ± 7.59 | 15.19 ± 14.04 | 31.71 ± 17.25 | 9.57 ± 12.05 | 24.24 ± 18.76 | 43.05 ± 21.97 |
| East Coast | 1015.93 | 8.19 ± 13.02 | 13.27 ± 8.84 | 10.52 ± 9.53 | 7.59 ± 8.82 | 13.07 ± 8.59 | 20.18 ± 14.01 | 7.57 ± 10.33 | 15.95 ± 12.45 | 30.76 ± 20.96 | 10.20 ± 11.76 | 23.33 ± 16.42 | 42.59 ± 29.57 |
| Ganga | 1147.34 | 10.08 ± 10.36 | 15.57 ± 9.25 | 15.73 ± 8.92 | 6.95 ± 7.78 | 15.05 ± 11.38 | 21.55 ± 15.19 | 8.73 ± 8.10 | 17.57 ± 16.09 | 32.37 ± 19.43 | 9.04 ± 9.75 | 22.65 ± 17.02 | 46.40 ± 27.44 |
| Godavari | 1152.89 | 14.11 ± 10.04 | 16.84 ± 11.70 | 17.34 ± 13.51 | 8.31 ± 7.19 | 17.38 ± 18.31 | 22.61 ± 16.37 | 10.60 ± 14.69 | 18.08 ± 25.07 | 34.41 ± 33.02 | 9.11 ± 16.89 | 24.14 ± 27.02 | 47.68 ± 45.24 |
| Indus | 525.11 | 13.26 ± 7.76 | 13.97 ± 8.22 | 12.56 ± 9.15 | 8.34 ± 9.03 | 13.45 ± 9.84 | 19.49 ± 11.55 | 11.66 ± 8.74 | 17.47 ± 12.42 | 33.94 ± 17.81 | 13.38 ± 8.87 | 23.32 ± 17.47 | 42.63 ± 23.93 |
| Krishna | 822.30 | 16.94 ± 14.17 | 19.15 ± 14.18 | 18.51 ± 19.63 | 11.33 ± 13.15 | 21.34 ± 23.29 | 26.50 ± 25.66 | 14.33 ± 16.98 | 23.61 ± 29.99 | 42.72 ± 43.57 | 14.38 ± 20.14 | 30.05 ± 37.47 | 53.98 ± 57.17 |
| Mahanadi | 1341.97 | 11.70 ± 10.18 | 16.46 ± 9.59 | 18.92 ± 10.16 | 5.49 ± 5.43 | 12.52 ± 10.70 | 19.73 ± 11.22 | 6.32 ± 9.77 | 12.14 ± 18.64 | 25.19 ± 20.50 | 5.46 ± 11.08 | 19.45 ± 17.62 | 38.62 ± 27.25 |
| Mahi | 880.62 | 25.68 ± 18.23 | 36.65 ± 23.40 | 32.59 ± 23.37 | 17.08 ± 19.71 | 37.21 ± 24.76 | 52.78 ± 30.10 | 25.43 ± 20.88 | 40.27 ± 39.92 | 67.94 ± 52.62 | 25.53 ± 22.98 | 53.21 ± 45.83 | 101.03 ± 84.23 |
| Narmada | 1126.08 | 18.94 ± 12.21 | 24.84 ± 14.13 | 23.70 ± 16.03 | 13.45 ± 12.69 | 26.38 ± 21.69 | 34.01 ± 19.75 | 16.38 ± 15.86 | 27.61 ± 31.84 | 45.64 ± 40.55 | 15.21 ± 18.76 | 35.76 ± 35.40 | 66.89 ± 59.08 |
| North East Coast | 1243.27 | 8.22 ± 8.22 | 11.93 ± 8.32 | 14.55 ± 7.47 | 1.94 ± 5.73 | 9.43 ± 8.36 | 13.50 ± 7.28 | 2.67 ± 6.68 | 5.99 ± 11.66 | 17.62 ± 14.22 | 2.38 ± 10.30 | 13.84 ± 12.43 | 29.46 ± 20.36 |
| Pennar | 777.74 | 12.81 ± 14.25 | 16.16 ± 9.20 | 14.26 ± 13.71 | 7.95 ± 11.26 | 14.82 ± 16.61 | 24.04 ± 21.49 | 8.89 ± 14.91 | 19.13 ± 20.90 | 37.09 ± 35.00 | 11.21 ± 16.27 | 25.83 ± 27.07 | 49.98 ± 40.28 |
| Sabarmati | 512.99 | 34.56 ± 25.48 | 43.63 ± 29.93 | 40.43 ± 25.48 | 24.41 ± 26.73 | 45.93 ± 27.36 | 73.83 ± 38.16 | 34.41 ± 24.35 | 51.81 ± 43.08 | 91.08 ± 56.88 | 36.31 ± 30.66 | 67.65 ± 50.14 | 130.00 ± 101.03 |
| South Coast | 1395.07 | 4.71 ± 12.14 | 12.36 ± 11.83 | 11.41 ± 11.70 | 5.45 ± 8.59 | 13.67 ± 11.96 | 17.76 ± 13.96 | 7.78 ± 9.42 | 12.85 ± 17.56 | 28.38 ± 21.43 | 6.83 ± 12.84 | 21.18 ± 19.97 | 38.46 ± 26.86 |
| Subarnarekha | 1428.76 | 7.27 ± 9.19 | 13.65 ± 9.85 | 14.28 ± 8.89 | 2.13 ± 7.57 | 9.55 ± 6.61 | 16.26 ± 13.28 | 2.84 ± 6.05 | 9.05 ± 11.08 | 21.69 ± 14.51 | 4.74 ± 8.02 | 16.54 ± 11.53 | 34.37 ± 17.33 |
| Tapi | 954.05 | 21.40 ± 12.85 | 26.07 ± 18.19 | 23.70 ± 18.32 | 15.31 ± 13.40 | 30.08 ± 25.86 | 37.87 ± 22.98 | 20.09 ± 19.91 | 32.17 ± 36.79 | 54.67 ± 49.96 | 18.26 ± 22.46 | 39.85 ± 41.76 | 76.12 ± 73.28 |
| West Coast | 2718.34 | 13.32 ± 11.58 | 16.27 ± 13.36 | 15.77 ± 15.55 | 9.49 ± 11.25 | 19.51 ± 17.69 | 22.88 ± 20.04 | 12.92 ± 12.99 | 18.82 ± 22.85 | 32.60 ± 31.04 | 12.94 ± 16.95 | 25.48 ± 29.57 | 42.83 ± 43.31 |

Table S6: Same as Table S5 but for mean annual maximum temperature (°C)

| **Basin** | **Historic mean** | **ssp126** | | | **ssp245** | | | **ssp370** | | | **ssp585** | | |
| --- | --- | --- | --- | --- | --- | --- | --- | --- | --- | --- | --- | --- | --- |
|  |  | **Near** | **Mid** | **Far** | **Near** | **Mid** | **Far** | **Near** | **Mid** | **Far** | **Near** | **Mid** | **Far** |
| Bhahmani | 32.08 | 0.43 ± 0.20 | 0.86 ± 0.28 | 0.90 ± 0.29 | 0.35 ± 0.30 | 1.05 ± 0.35 | 1.43 ± 0.43 | 0.30 ± 0.36 | 1.07 ± 0.46 | 2.04 ± 0.66 | 0.35 ± 0.40 | 1.52 ± 0.48 | 2.88 ± 0.78 |
| Brahmaputra | 18.41 | 0.81 ± 0.17 | 1.23 ± 0.27 | 1.24 ± 0.36 | 0.81 ± 0.15 | 1.56 ± 0.30 | 2.05 ± 0.40 | 0.80 ± 0.24 | 1.78 ± 0.33 | 2.93 ± 0.51 | 0.95 ± 0.23 | 2.25 ± 0.35 | 3.79 ± 0.59 |
| Cauvery | 29.78 | 0.51 ± 0.27 | 0.80 ± 0.34 | 0.84 ± 0.36 | 0.48 ± 0.33 | 1.05 ± 0.41 | 1.36 ± 0.54 | 0.47 ± 0.35 | 1.20 ± 0.49 | 2.01 ± 0.73 | 0.53 ± 0.34 | 1.50 ± 0.54 | 2.57 ± 0.92 |
| East Coast | 32.75 | 0.56 ± 0.32 | 0.93 ± 0.37 | 0.99 ± 0.36 | 0.52 ± 0.39 | 1.23 ± 0.46 | 1.57 ± 0.59 | 0.49 ± 0.48 | 1.36 ± 0.62 | 2.32 ± 0.86 | 0.55 ± 0.46 | 1.72 ± 0.66 | 3.01 ± 1.03 |
| Ganga | 28.39 | 0.62 ± 0.28 | 1.11 ± 0.39 | 1.17 ± 0.42 | 0.55 ± 0.27 | 1.37 ± 0.42 | 1.85 ± 0.51 | 0.45 ± 0.40 | 1.38 ± 0.47 | 2.49 ± 0.67 | 0.60 ± 0.40 | 1.92 ± 0.55 | 3.47 ± 0.84 |
| Godavari | 32.89 | 0.42 ± 0.28 | 0.88 ± 0.44 | 0.88 ± 0.43 | 0.45 ± 0.35 | 1.09 ± 0.56 | 1.49 ± 0.63 | 0.33 ± 0.44 | 1.16 ± 0.61 | 2.14 ± 0.88 | 0.47 ± 0.43 | 1.57 ± 0.66 | 2.91 ± 1.09 |
| Indus | 25.32 | 0.93 ± 0.29 | 1.39 ± 0.45 | 1.43 ± 0.49 | 0.98 ± 0.27 | 1.84 ± 0.48 | 2.41 ± 0.62 | 0.94 ± 0.34 | 2.10 ± 0.59 | 3.38 ± 0.81 | 1.10 ± 0.35 | 2.60 ± 0.61 | 4.40 ± 1.04 |
| Krishna | 32.11 | 0.43 ± 0.29 | 0.82 ± 0.41 | 0.83 ± 0.42 | 0.44 ± 0.34 | 1.03 ± 0.53 | 1.38 ± 0.62 | 0.37 ± 0.39 | 1.14 ± 0.57 | 2.00 ± 0.84 | 0.46 ± 0.39 | 1.47 ± 0.63 | 2.65 ± 1.05 |
| Mahanadi | 32.59 | 0.43 ± 0.20 | 0.88 ± 0.36 | 0.90 ± 0.34 | 0.41 ± 0.32 | 1.12 ± 0.41 | 1.50 ± 0.49 | 0.31 ± 0.41 | 1.15 ± 0.51 | 2.17 ± 0.72 | 0.42 ± 0.42 | 1.59 ± 0.54 | 3.00 ± 0.88 |
| Mahi | 33.21 | 0.53 ± 0.41 | 0.94 ± 0.58 | 0.99 ± 0.60 | 0.56 ± 0.43 | 1.20 ± 0.70 | 1.60 ± 0.86 | 0.39 ± 0.52 | 1.29 ± 0.75 | 2.29 ± 1.04 | 0.58 ± 0.51 | 1.70 ± 0.83 | 3.03 ± 1.29 |
| Narmada | 32.82 | 0.48 ± 0.37 | 0.94 ± 0.53 | 0.99 ± 0.52 | 0.49 ± 0.39 | 1.19 ± 0.65 | 1.61 ± 0.74 | 0.33 ± 0.52 | 1.22 ± 0.71 | 2.28 ± 0.97 | 0.53 ± 0.49 | 1.69 ± 0.74 | 3.11 ± 1.16 |
| North East Coast | 31.61 | 0.38 ± 0.17 | 0.78 ± 0.28 | 0.77 ± 0.27 | 0.39 ± 0.27 | 0.99 ± 0.32 | 1.32 ± 0.38 | 0.33 ± 0.30 | 1.07 ± 0.40 | 1.96 ± 0.56 | 0.40 ± 0.30 | 1.43 ± 0.43 | 2.65 ± 0.73 |
| Pennar | 32.73 | 0.47 ± 0.30 | 0.86 ± 0.39 | 0.89 ± 0.40 | 0.46 ± 0.36 | 1.12 ± 0.49 | 1.45 ± 0.61 | 0.40 ± 0.44 | 1.22 ± 0.60 | 2.13 ± 0.86 | 0.47 ± 0.41 | 1.57 ± 0.65 | 2.84 ± 1.07 |
| Sabarmati | 33.09 | 0.62 ± 0.31 | 1.01 ± 0.47 | 1.05 ± 0.51 | 0.65 ± 0.32 | 1.33 ± 0.53 | 1.73 ± 0.67 | 0.55 ± 0.37 | 1.45 ± 0.57 | 2.46 ± 0.79 | 0.70 ± 0.40 | 1.84 ± 0.63 | 3.17 ± 0.99 |
| South Coast | 31.53 | 0.62 ± 0.25 | 0.90 ± 0.33 | 0.95 ± 0.36 | 0.59 ± 0.30 | 1.20 ± 0.40 | 1.56 ± 0.52 | 0.59 ± 0.30 | 1.43 ± 0.46 | 2.32 ± 0.68 | 0.67 ± 0.32 | 1.73 ± 0.55 | 2.90 ± 0.88 |
| Subarnarekha | 31.66 | 0.44 ± 0.22 | 0.90 ± 0.27 | 0.97 ± 0.30 | 0.27 ± 0.32 | 1.03 ± 0.34 | 1.45 ± 0.45 | 0.23 ± 0.37 | 0.96 ± 0.47 | 1.96 ± 0.68 | 0.29 ± 0.46 | 1.51 ± 0.52 | 2.87 ± 0.79 |
| Tapi | 33.91 | 0.46 ± 0.38 | 0.92 ± 0.51 | 0.95 ± 0.52 | 0.49 ± 0.40 | 1.12 ± 0.69 | 1.54 ± 0.77 | 0.33 ± 0.49 | 1.19 ± 0.71 | 2.17 ± 0.99 | 0.53 ± 0.46 | 1.60 ± 0.75 | 2.93 ± 1.19 |
| West Coast | 31.43 | 0.59 ± 0.29 | 0.96 ± 0.44 | 0.98 ± 0.46 | 0.59 ± 0.30 | 1.22 ± 0.53 | 1.63 ± 0.65 | 0.55 ± 0.31 | 1.40 ± 0.52 | 2.37 ± 0.82 | 0.66 ± 0.34 | 1.75 ± 0.61 | 3.02 ± 1.04 |

Table S7: Same as Table S5 but for mean annual minimum temperature (°C)

| **Basin** | **Historic mean** | **ssp126** | | | **ssp245** | | | **ssp370** | | | | **ssp585** | | |
| --- | --- | --- | --- | --- | --- | --- | --- | --- | --- | --- | --- | --- | --- | --- |
|  |  | **Near** | **Mid** | **Far** | **Near** | **Mid** | **Far** | **Near** | **Mid** | **Far** | **Near** | | **Mid** | **Far** |
| Bhahmani | 20.85 | 0.64 ± 0.17 | 1.06 ± 0.33 | 1.09 ± 0.34 | 0.61 ± 0.30 | 1.40 ± 0.39 | 1.85 ± 0.43 | 0.60 ± 0.34 | 1.63 ± 0.47 | 2.83 ± 0.61 | 0.67 ± 0.38 | | 2.07 ± 0.46 | 3.71 ± 0.67 |
| Brahmaputra | 6.42 | 1.04 ± 0.25 | 1.47 ± 0.40 | 1.44 ± 0.46 | 1.07 ± 0.25 | 1.97 ± 0.44 | 2.55 ± 0.51 | 1.13 ± 0.36 | 2.38 ± 0.55 | 3.81 ± 0.74 | 1.20 ± 0.35 | | 2.77 ± 0.54 | 4.67 ± 0.94 |
| Cauvery | 19.63 | 0.68 ± 0.26 | 0.98 ± 0.42 | 0.99 ± 0.41 | 0.70 ± 0.27 | 1.38 ± 0.46 | 1.75 ± 0.59 | 0.74 ± 0.28 | 1.66 ± 0.49 | 2.64 ± 0.79 | 0.81 ± 0.29 | | 1.99 ± 0.57 | 3.25 ± 1.00 |
| East Coast | 22.61 | 0.67 ± 0.27 | 1.01 ± 0.45 | 1.02 ± 0.44 | 0.68 ± 0.29 | 1.39 ± 0.50 | 1.76 ± 0.61 | 0.72 ± 0.33 | 1.67 ± 0.53 | 2.66 ± 0.80 | 0.78 ± 0.35 | | 2.01 ± 0.59 | 3.27 ± 1.01 |
| Ganga | 15.82 | 0.90 ± 0.31 | 1.32 ± 0.46 | 1.34 ± 0.50 | 0.86 ± 0.36 | 1.76 ± 0.53 | 2.32 ± 0.65 | 0.84 ± 0.41 | 2.04 ± 0.69 | 3.42 ± 0.96 | 0.99 ± 0.44 | | 2.56 ± 0.70 | 4.42 ± 1.12 |
| Godavari | 20.75 | 0.74 ± 0.33 | 1.21 ± 0.50 | 1.21 ± 0.50 | 0.75 ± 0.38 | 1.62 ± 0.57 | 2.11 ± 0.68 | 0.71 ± 0.40 | 1.86 ± 0.65 | 3.15 ± 0.90 | 0.87 ± 0.42 | | 2.35 ± 0.70 | 4.05 ± 1.08 |
| Indus | 11.95 | 1.16 ± 0.36 | 1.55 ± 0.50 | 1.52 ± 0.55 | 1.18 ± 0.36 | 2.12 ± 0.58 | 2.73 ± 0.75 | 1.19 ± 0.43 | 2.51 ± 0.77 | 4.04 ± 1.10 | 1.37 ± 0.45 | | 3.07 ± 0.82 | 5.10 ± 1.35 |
| Krishna | 20.68 | 0.75 ± 0.32 | 1.17 ± 0.50 | 1.17 ± 0.51 | 0.78 ± 0.33 | 1.61 ± 0.54 | 2.09 ± 0.65 | 0.76 ± 0.35 | 1.84 ± 0.62 | 3.01 ± 0.89 | 0.88 ± 0.38 | | 2.27 ± 0.68 | 3.77 ± 1.07 |
| Mahanadi | 20.78 | 0.69 ± 0.22 | 1.12 ± 0.40 | 1.14 ± 0.41 | 0.67 ± 0.33 | 1.50 ± 0.46 | 1.95 ± 0.53 | 0.64 ± 0.37 | 1.75 ± 0.52 | 3.01 ± 0.69 | 0.75 ± 0.39 | | 2.21 ± 0.55 | 3.91 ± 0.81 |
| Mahi | 19.85 | 0.93 ± 0.47 | 1.32 ± 0.60 | 1.36 ± 0.67 | 0.92 ± 0.46 | 1.77 ± 0.70 | 2.34 ± 0.92 | 0.84 ± 0.44 | 2.06 ± 0.79 | 3.46 ± 1.13 | 1.06 ± 0.53 | | 2.59 ± 0.93 | 4.39 ± 1.46 |
| Narmada | 19.50 | 0.85 ± 0.41 | 1.29 ± 0.56 | 1.32 ± 0.59 | 0.83 ± 0.43 | 1.72 ± 0.64 | 2.27 ± 0.81 | 0.75 ± 0.43 | 1.99 ± 0.74 | 3.42 ± 1.04 | 0.96 ± 0.48 | | 2.54 ± 0.80 | 4.41 ± 1.27 |
| North East Coast | 21.70 | 0.61 ± 0.19 | 1.04 ± 0.39 | 1.04 ± 0.38 | 0.62 ± 0.29 | 1.37 ± 0.43 | 1.78 ± 0.48 | 0.61 ± 0.32 | 1.61 ± 0.48 | 2.74 ± 0.63 | 0.68 ± 0.34 | | 2.01 ± 0.51 | 3.50 ± 0.75 |
| Pennar | 21.92 | 0.64 ± 0.28 | 1.01 ± 0.45 | 1.01 ± 0.45 | 0.66 ± 0.29 | 1.40 ± 0.48 | 1.79 ± 0.58 | 0.68 ± 0.33 | 1.65 ± 0.53 | 2.67 ± 0.77 | 0.76 ± 0.33 | | 2.02 ± 0.57 | 3.35 ± 0.96 |
| Sabarmati | 20.25 | 1.08 ± 0.40 | 1.49 ± 0.55 | 1.51 ± 0.65 | 1.07 ± 0.41 | 2.02 ± 0.65 | 2.62 ± 0.85 | 1.05 ± 0.43 | 2.33 ± 0.78 | 3.82 ± 1.11 | 1.23 ± 0.51 | | 2.89 ± 0.88 | 4.76 ± 1.38 |
| South Coast | 22.95 | 0.76 ± 0.27 | 1.09 ± 0.45 | 1.09 ± 0.45 | 0.77 ± 0.30 | 1.49 ± 0.51 | 1.89 ± 0.64 | 0.80 ± 0.31 | 1.81 ± 0.54 | 2.88 ± 0.81 | 0.90 ± 0.33 | | 2.17 ± 0.61 | 3.56 ± 0.93 |
| Subarnarekha | 20.67 | 0.63 ± 0.18 | 1.05 ± 0.29 | 1.09 ± 0.31 | 0.58 ± 0.32 | 1.35 ± 0.37 | 1.80 ± 0.43 | 0.55 ± 0.36 | 1.51 ± 0.51 | 2.67 ± 0.65 | 0.64 ± 0.42 | | 2.00 ± 0.45 | 3.58 ± 0.67 |
| Tapi | 20.77 | 0.91 ± 0.44 | 1.37 ± 0.57 | 1.39 ± 0.61 | 0.91 ± 0.43 | 1.83 ± 0.67 | 2.42 ± 0.84 | 0.85 ± 0.42 | 2.15 ± 0.77 | 3.57 ± 1.10 | 1.07 ± 0.48 | | 2.69 ± 0.85 | 4.52 ± 1.30 |
| West Coast | 21.44 | 1.01 ± 0.40 | 1.47 ± 0.61 | 1.49 ± 0.64 | 1.04 ± 0.40 | 2.00 ± 0.64 | 2.59 ± 0.75 | 1.04 ± 0.37 | 2.29 ± 0.67 | 3.64 ± 0.93 | 1.18 ± 0.44 | | 2.79 ± 0.75 | 4.46 ± 1.06 |
